# Exploration of alternative microfiltration modalities for the harvest and clarification of diverse recombinant proteins from high-density *E. coli* culture and lysate using hollow fibre, flat sheet cassette, and vibro membrane filtration technologies

**DOI:** 10.1093/jimb/kuaf008

**Published:** 2025-04-04

**Authors:** Jennifer Reid, Joyce Ni, Airong Chen, Patricia Gomes, Andrew Szto, Analyn Yu, Angela Luo, Belinda Kong, Calvin Adams, Neveathan Jeyachandran, Anumta Amir, Xavier Teixeira, Tao Yuan, Cédric Charretier

**Affiliations:** Global Bioprocess Development - Drug Substance Development, Sanofi Vaccines, Toronto M2R 3T4 ON, Canada; Global Bioprocess Development - Drug Substance Development, Sanofi Vaccines, Toronto M2R 3T4 ON, Canada; Global Bioprocess Development - Drug Substance Development, Sanofi Vaccines, Toronto M2R 3T4 ON, Canada; Global Bioprocess Development - Drug Substance Development, Sanofi Vaccines, Toronto M2R 3T4 ON, Canada; Global Bioprocess Development - Drug Substance Development, Sanofi Vaccines, Toronto M2R 3T4 ON, Canada; Global Bioprocess Development - Drug Substance Development, Sanofi Vaccines, Toronto M2R 3T4 ON, Canada; Global Bioprocess Development - Drug Substance Development, Sanofi Vaccines, Toronto M2R 3T4 ON, Canada; Global Bioprocess Development - Drug Substance Development, Sanofi Vaccines, Toronto M2R 3T4 ON, Canada; Global Bioprocess Development - Drug Substance Development, Sanofi Vaccines, Toronto M2R 3T4 ON, Canada; Global Bioprocess Development - Drug Substance Development, Sanofi Vaccines, Toronto M2R 3T4 ON, Canada; Global Bioprocess Development - Drug Substance Development, Sanofi Vaccines, Toronto M2R 3T4 ON, Canada; Global Bioprocess Development - Drug Substance Development, Sanofi Vaccines, Marcy-l'Étoile, 69280, France; Global Bioprocess Development - Drug Substance Development, Sanofi Vaccines, Toronto M2R 3T4 ON, Canada; Global Bioprocess Development - Drug Substance Development, Sanofi Vaccines, Marcy-l'Étoile, 69280, France

**Keywords:** Clarification, Midstream, *E. coli*, Bioprocessing, Fermentation

## Abstract

Industrial bioprocess optimization has significantly increased the productivity of biomass and biologics in upstream production. Such process improvement in fermentation often translates to challenges in recovering intracellularly expressed recombinant proteins due to increased matrix complexity, resulting in a higher performance burden in midstream. Tangential flow filtration (TFF) is a popular industry standard for buffer exchange and protein separation from cellular debris. However, due to variations in the physicochemical properties of recombinant proteins, solutions for *E. coli*-based protein clarification remain challenging and often necessitate extensive exploration and process optimization. With growing options in filtration-based technologies, the identification of a near-universal clarification platform is desirable to accelerate bioprocess development overall. In this study, three TFF modalities, hollow fibre (HF), flat-sheet cassette (CAS), and vibro membrane filtration (VMF), were assessed in parallel to evaluate their clarification performance for three *E. coli* recombinant proteins with different biochemical properties. Reverse phase liquid chromatography data showed target protein recovery was uniformly higher for VMF than HF at equivalent loading. Cell density and lysate protein load were comparable for HF and VMF, and lower for CAS. These results support the choice of VMF and HF as easily optimized and operated TFF modalities for clarification of recombinant protein from complex crude bacterial matrix, where either can be efficiently performed with ease and minimum supervision. Both TFF applications were successfully demonstrated in primary cell harvest, cell wash and cell lysate clarification, for *E. coli*-based recombinant proteins.

**One-Sentence Summary:**

High-density *E. coli* microfiltration and lysate clarification were tested for three diverse recombinant proteins, where hollow fibre and vibro membrane filtration outperformed flat sheet cassette in terms of process time, suspended solid loading, and target protein recovery.

## Graphical Abstract

**Graphical Abstract:**
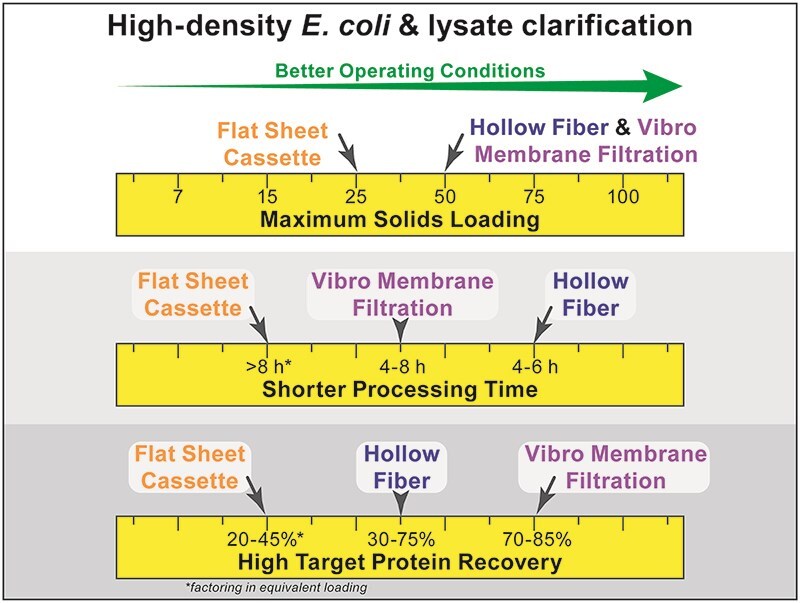

## Introduction

Process intensification has significantly increased the productivity of biologics of interest from stirred tank reactors in upstream production (Müller et al., 2022). Process improvement to attain high-density cultures often translates to challenges in recovering biologics due to increased biomass and matrix complexity, resulting in a higher performance burden in midstream.

Midstream, also referred to as primary recovery, is sometimes neglected during process development, while at the same time having a direct impact on downstream purification process performance (Besnard et al., 2016). Midstream comprises the unit operations between upstream production in bioreactors, and downstream purification using chromatography. It requires the removal of solids (i.e. cell biomass and debris) and is different in scope to traditional downstream TFF ultrafiltration/diafiltration (UF/DF), which typically operates in the range of 0.02–0.05 µm pore sizes. Various clarification technologies are better suited to viral, whole cell, and recombinant protein biologics. Despite upstream process intensification, including perfusion, efforts for monoclonal antibody production from Chinese hamster ovary (CHO) cell culture remain sufficiently low enough in density that it can be depth filtered and sterile filtered (i.e. normal flow filtration, NFF) without significant process optimization (Besnard et al., 2016; Xu et al., 2020). CHO cell density has been reported as ‘*0.00666 mL pellet volume per mL culture volume and per viable cell concentration of 10^6^ cells/mL*’, which translates to approximately 7 g cell wet weight (CWW) per litre of culture (LoC) when there are 1 × 10^6^ cells/mL (Müller et al., 2022). Similarly, eukaryotic insect cells are used in the production of viral particles and can be clarified with depth filters or hollow fibre (HF) membranes as the cell density is quite low (2 × 10^6^ cells/mL, Besnard et al., 2016; Negrete et al., 2014). In cases of eukaryotic cell culture for the production of adeno-associated viruses (Jiang et al., 2023), and the aforementioned viral particles and monoclonal antibodies, clarification using simple NFF technologies is warranted due to its simplicity of use and low solid content from production bioreactors.

Manufacture of microbial enzymes and other recombinant proteins has broad importance in food, detergent, pharmaceutical, and paper industries (Raveendran et al., 2018), with a net worth exceeding $6 billion USD in 2020 (Okpara et al., 2022). In contrast to mammalian cell culture, high-density microbial cultures, such as *Escherichia coli* (*E. coli)*, produce >100–150 g CWW/LoC (Bailey et al., 1997), approximately 20 times greater density compared to typical CHO processes. Reviewed microbial clarification methods generally consist of extracellular biologics (Besnard et al., 2016), and as such are similar in principle to the simple removal of solids for mammalian cell culture, where the product is largely contained in the spent media. Additionally, very low microbial density can permit depth filtration, that is optical density at 600 nm (OD_600_) of 4 (Allahghadry et al., 2022), which is useful if the product is expressed extracellularly. To recover the intracellularly expressed recombinant protein of interest from high-density *E. coli* cultures, cells are typically harvested, followed by cell concentration and cell wash through buffer exchange. In this case, it is important to avoid undesired premature lysis during cell concentration and replacement of spent media with a stabilizing buffer. Subsequently, cell disruption is performed to release the recombinant protein of interest. The cell lysate at this stage contains a mixture of cell debris, DNA, and host cell proteins (HCPs). Cell lysate clarification to recover a biological product from such a highly dense, viscous, and complex mixture has a greater propensity to foul membranes used for clarification, and is therefore inherently more difficult than clarifying extracellular components from spent media.

Tangential flow filtration (TFF) is a popular industry standard for buffer exchange and protein separation from cellular debris. However, due to variations in the physicochemical properties of recombinant proteins, solutions for high-density *E. coli*-based protein clarification remain challenging and often necessitate extensive exploration and process optimization. Traditional clarification technologies include membrane filtration (microfiltration, TFF), centrifugation, and depth filtration (i.e. NFF) (Besnard et al., 2016). For simplicity during process development, lysate clarification is often performed by centrifugation and depth filtration. However, TFF is also popular for microfiltration, with membrane cut-offs in the range of 0.1–0.65 μm used to retain cells and debris (Besnard et al., 2016). With growing options in TFF-based technologies, the identification of a near-universal clarification platform is desirable to accelerate bioprocess development overall. Highly automated TFF systems also minimize supervision and work force requirements, thus making TFF an attractive platform technology.

However, TFF comes with well-known process constraints such as membrane fouling and solution viscosity. Membrane fouling is the blockage of pores, pore constriction, and caking of the membrane surface by soluble or suspended matter, all of which can reduce permeate flux over time (Duclos-Orsello et al., 2006). This can extend processing time and limit the solid loading per membrane surface area, both acting to increase the cost of goods. Reviews have reported that HF TFF has higher solid loading capacities as compared to flat-sheet cassette TFF (CAS) (Besnard et al., 2016). A simple solution to reduce membrane fouling is to dilute the lysate material and reduce the solid load on the membrane, as previously reported (Bailey et al., 1997). However, this may exacerbate the challenges of industrial scale-up due to operation complexity involving larger volumes of buffers and longer process time. HF have been reported to be nonlinear in scaling (Besnard et al., 2016) compared to other membrane configurations such as CAS, since decrease in trans-membrane pressure (TMP) along the flow path is more prominent for HF due to significant pressure drops along the narrow fibre lumen (Li et al., 2022). Therefore, longer HF membranes may be more significantly affected by rheology and non-Newtonian effects of shear thinning (Zhang et al., 2022).

A relatively new TFF technology emerging in the food and biologics industry is vibro membrane filtration (VMF), which has been used for microfiltration to concentrate microalgae (Grácio et al., 2024; Six et al., 2024), and UF of milk (Coşkun et al., 2023). This technology purportedly supports a low-pressure system, linear scale-up, and high solid loading. Interestingly, it appears that the solid loading capacity of HF has not been directly compared to newer modalities such as VMF, despite reports of concentrating microbial cells to a slurry consistency with VMF (Grácio et al., 2024; Six et al., 2024). Furthermore, the authors are not aware of industrialized pharmaceutical processes having assessed VMF, especially for microbial lysate clarification.

In this study, three TFF modalities (HF, VMF, and CAS) were assessed in parallel to evaluate their microfiltration and clarification performance in the recovery of three recombinant proteins, each with different biochemical properties, from high-density *E. coli* cultures. The TFF systems were fully automated, allowing for overnight processing (if required) with minimal downtime. The three TFF modalities were evaluated for two unit operations: primary cell harvest (concentration and buffer exchange) and cell lysate clarification. Different operating conditions were developed, which were suitable for each TFF modality and for the particular needs of both unit operations.

## Materials and Methods

### Protein Production

Three recombinant proteins were evaluated: acidic monomer (AM), basic monomer (BM), and acidic oligomer (AO). AM is a monomer with a molecular weight between 40 and 60 kDa and pI ≈ 5–6. BM is a monomer with a molecular weight of 20–40 kDa and pI ≈ 10–11. AO is an oligomer with a molecular weight of 90–120 kDa and pI ≈ 5–6. All three proteins are soluble and are expressed by an isopropyl β-D-1-thiogalactopyranoside (IPTG)-inducible plasmid transformed into *E. coli* BL21(DE3). Fermentation details have been previously described (Farrell et al., 2015). In brief, antibiotic-free and chemically defined media are used in preculture shake flasks and during fermentation. Two-litre and 20-L Biostat (both Sartorius) fermenters were used in this study. A batch phase was followed by an overnight carbon-limited fed-batch process that was initiated by a dissolved oxygen (DO) spike as previously described (Korz et al., 1995). Culture optical density at 600 nm (OD_600_) was measured offline using a spectrophotometer and IPTG was used as the induction trigger. Crude harvest characteristics are displayed in Table 1. Optical density at 600 nm (OD_600_) was measured with a BioMate 160 UV Visible spectrophotometer (ThermoFisher). Turbidity was measured with a 2100Q turbidimeter (Hach) according to the manufacturer's recommendations. Solution particle size measurements were performed with a Mastersizer 2000 (Malvern Panalytical) according to the manufacturer's recommendations. Solution viscosity was measured at ambient temperature with a microVisc (Rheosense) using the 24HA05100475 cassette according to manufacturer's recommendations.

**Table 1.** Cell Wash Microfiltration Characteristics

Cell wet weight (g/LoC)	OD_600_	Turbidity (NTU)	PSD_10_ (µm)	PSD_50_ (µm)	PSD_90_ (µm)	Viscosity at 2,000 s^−1^ (cP)	Viscosity at 4,000 s^−1^ (cP)	Viscosity at 6,000 s^−1^ (cP)
113 ± 4	76 ± 4	31 600 ± 7 400	0.624	0.956	1.500	1.57	1.58	1.58

PSD_10_, PSD_50_, and PSD_90_ indicate that 10%, 50%, and 90% respectively, of the total number of measured particles were smaller than this size. Three independent fermentation runs were produced (one per recombinant protein), and the average ± standard deviation is shown. Particle size and viscosity were tested on one lot, therefore single values are reported.

### Homogenization

Cell disruption was achieved using high pressure homogenization (EmulsiFlex-C55, Avestin), with 3 cycles at 900 bar. Homogenate was diluted (as required) back to the original crude harvest volume, aliquoted in multiple containers, and stored at −80°C. Frozen aliquots were thawed in an ambient water bath for approximately 3 hr on the same day they were used for clarification. Thawed homogenate was mixed with constant stirring prior to use. Homogenate containing AM protein was tested fresh (same day as homogenization) and thawed to assess comparability for clarification. Cell lysate characteristics are displayed in Table 2 using the process analytical tools described in *Protein Production*.

**Table 2.** *E. coli* Lysate Characteristics

Target protein	^a^Cell wet weight (g/LoC)	OD_600_	Turbidity (NTU)	PSD_10_ (µm)	PSD_50_ (µm)	PSD_90_ (µm)	Viscosity at 4,000 s^−1^ (cP)	Viscosity at 6,000 s^−1^ (cP)	Viscosity at 8,000 s^−1^ (cP)
AM	110	3.7	2 940	0.088	0.227	1.025	1.29	1.33	1.28
BM	81	5.6	3 380	0.080	0.194	1.210	low: 1.67	low: 1.55	low: 1.60
							high: 1.73	high: 1.74	high: 1.64
AO	117	5.4	4 020	0.574	4.492	10.922	2.53	2.25	2.12

PSD_10_, PSD_50_, and PSD_90_ indicate that 10%, 50%, and 90% respectively, of the total number of measured particles were smaller than this size.

a Cell wet weight (g/LoC) prior to high pressure homogenization, all other measurements performed after homogenization. For BM protein, low and high refers to 100 and 500 mM NaCl in the buffer, respectively. Abbreviations: acidic monomer (AM), basic monomer (BM), and acidic oligomer (AO).

### Operating Parameters for Cell Wash TFF

The starting material used in cell wash TFF studies consisted of *E. coli* grown as described above to a density of 80–100 OD_600_ suspended in spent media. The material was used fresh, directly after harvesting from a fermenter. Bovine serum albumin (BSA, Sigma-Aldrich) was spiked-in to a final concentration of 0.4 g/L, which was used for SDS-PAGE analysis of membrane transmission. 20 mM Tris HCl pH 8.5 buffer or 50 mM Sodium Citrate pH 6.0 buffer was used for cell wash DF. No differences were observed between *E. coli* from either condition (Table 1). The ambient operating temperature was 20 ± 2°C.

### Operating Parameters for Lysate Clarification TFF

Cell lysate was thawed in an ambient-temperature waterbath on the morning of use, except for one experiment where fresh lysate was used directly following homogenization (indicated in Fig. S1B and C). Homogenate clarification was performed with HF, VMF, and CAS to achieve 1.5× concentration from the harvest culture volume and 3-diavolume filtration. The operating parameters tested are described as follows: all modalities operated with permeate control as depicted in Fig. 3a, with the shear (HF only), membrane loading (LoC/m^2^), and pore size (750 kDa, 800 kDa, or 0.2 µm) indicated in Fig. 3b for each material. The DF buffers used for *E. coli* lysate containing the target proteins were 20–50 mM Tris HCl or sodium citrate, at a pH between 6.0 and 9.0 based on process development optimization. Five hundred millimolar NaCl was added to lysate containing BM proteins (unless otherwise stated in Fig. S1A). The ambient operating temperature was 20 ± 2°C.

### HF TFF Preparation and Cleaning

HF membranes were operated using the 8-channel Ambr Crossflow system fitted with 8 Ambr® CF Adapter Kit SC50’s (Sartorius Stedim). The following two HF membranes were used for cell wash studies: termed ‘750 kDa’ (750 kDa pore size, modified polyether sulfone (mPES), 13 cm^2^, 1 mm fibre diameter, catalogue # C02-E750-10-N, Repligen) and ‘0.2 µm’ (0.2 µm pore size, mPES, 13 cm^2^, 1 mm fibre diameter, catalogue # C02-P20U-10-N, Repligen). The following two HF membranes were used for lysate clarification studies: termed ‘750 kDa’ (750 kDa pore size, mPES, 20 cm^2^, 0.5 mm fibre diameter, catalogue # C02-E750-05-N, Repligen), and ‘0.2 µm’ (0.2 µm pore size, mPES, 28 cm^2^, 0.5 mm fibre diameter, catalogue # C02-P20U-05-N, Repligen). The Ambr Crossflow and HF were flushed with ultra-pure water, and then flushed with buffer using default settings. HF were drained just prior to use. Experimental run parameters are described in ‘*Operating Parameters for Cell Wash TFF*’ and ‘*Operating Parameters for Lysate Clarification TFF’*. After run completion, the Ambr Crossflow was appropriately sanitized according to the manufacturer's recommendation and the HF was discarded after single use.

### Flat-sheet Cassette (CAS) TFF Preparation and Cleaning

CAS were operated using the Sartoflow Smart Crossflow system (Sartorius Stedim). Sartocon Slice membranes [regenerated cellulose (RC) Hydrosart, Sartorius Stedim] with 0.2 µm pore sizes were used, with a membrane surface area of 200 cm^2^. The Sartoflow and CAS were flushed with appropriate volumes of ultra-pure water and buffer according to supplier recommendation. Experimental run parameters are described in ‘*Operating Parameters for Cell Wash TFF*’ and ‘*Operating Parameters for Lysate Clarification TFF’*. Material was recirculated for 5 min prior to starting permeate flow, and gradually increased if permitted. After run completion, the system was appropriately sanitized according to the manufacturer's recommendation. Water flux was measured to assess membrane integrity and membranes were discarded if significantly diminished and not reused.

### Vibro Membrane TFF (VMF) Preparation and Cleaning

The Vibro-Lab280 (SANI Membranes) was operated using the KrosFlo KR2i TFF system (Repligen). Lab280 Cartridge Membranes of 800 kDa (polyvinylidene fluoride, PVDF) or 0.2 µm (polyether sulfone, PES) pore sizes were used, with a membrane surface area of 280 cm^2^. During flushing and operation, vibration was turned on. The VMF cartridge was drained of 20% isopropyl alcohol (IPA) storage solution, flushed with 3 L of ultra-pure water, and then flushed with 3 L of buffer at 200 mL/min, with manual retentate back-pressure used to increase permeate flow. The VMF cartridge was drained just prior to use. Experimental run parameters are described in ‘*Operating Parameters for Cell Wash TFF*’ and ‘*Operating Parameters for Clarification TFF’*. After run completion, the system was appropriately sanitized according to the manufacturer's recommendation. Water flux was measured to assess membrane integrity and membranes were discarded if significantly diminished and not reused.

### SDS-PAGE

Reducing SDS-PAGE was performed using NuPAGE® pre-cast 10% or 4–12% Bis-Tris gels, using 1× MOPS buffer as the SDS-PAGE running buffer, NuPAGE® LDS Sample Buffer, and NuPAGE® Sample Reducing Agent. Samples were heated up to 70°C for 10 min, then 10–20 µL were loaded onto the gel and proteins were separated using the Xcell SureLock Mini-Cell system (Invitrogen), in reducing conditions. Molecular weight standards, Precision Plus Protein Standard (BioRad) or SeeBlue Plus 2 Pre-stained Standard (Invitrogen), were loaded onto each gel for reference. Gels were stained with InstantBlue or ReadyBlue Protein Gel Stain (both from Sigma-Aldrich) and de-stained with ultra-pure water. Gel images were acquired on a GS900 Densitometer (BioRad) scanner and processed with ImageLab.

### Reverse Phase Liquid Chromatography (RP-LC)

Samples were centrifuged twice by 20,800× *g* for 5 min at ambient temperature with the supernatant transferred twice in series to remove any pelleted material and transferred into a glass high performance liquid chromatography (HPLC) vial. Samples were injected into a BioResolve RP mAB Polyphenyl 450 Å, 2.7 µm, 2.1 × 150 mm column (Waters) on an Infinity II 1260 HPLC (Agilent) with a Diode Array Detector (DAD). The standard curve was constructed using the purified protein of interest. Samples were separated using a 0.1% Trifluoroacetic Acid (TFA) in water/0.1% TFA in Acetonitrile (ACN) gradient. Both needle wash and purge solvent were 50% ACN in water (v/v). Detection was performed at 215 nm and integration performed using ChemStation (Agilent). The target protein concentration was calculated using linear regressing analysis of the standard curve.

### Endotoxin and Protein Quantitation

The bicinchoninic acid assay (BCA) protein assay kit (Thermo Fisher Scientific) was used for total protein concentration. Serial dilutions of duplicates were placed on a 96-well microplate (catalogue # 167 008, Thermo Scientific). After incubating samples according to the manufacturer's guidelines, the absorbance was measured at 570 nm. Readings were accepted if they achieved a sample coefficient of variation, as a percent of the mean, of (%CV) ≤15% total protein concentration, and were quantified relative to a BSA standard curve ranging from 0 to 2000 µg/mL. Endotoxin was detected using Endosafe Nexgen PTS System (Charles River), with 10–0.1 EU/mL or 1–0.01 EU/mL cartridges. Readings were accepted if they achieved Sample %CV and Spike %CV ≤10% and spike recovery was 50–200%. Serial dilutions with LAL reagent water were performed initially, and the final dilutions were performed in phosphate buffered saline to the desired concentration.

### Statistics

**Fig. 1.**
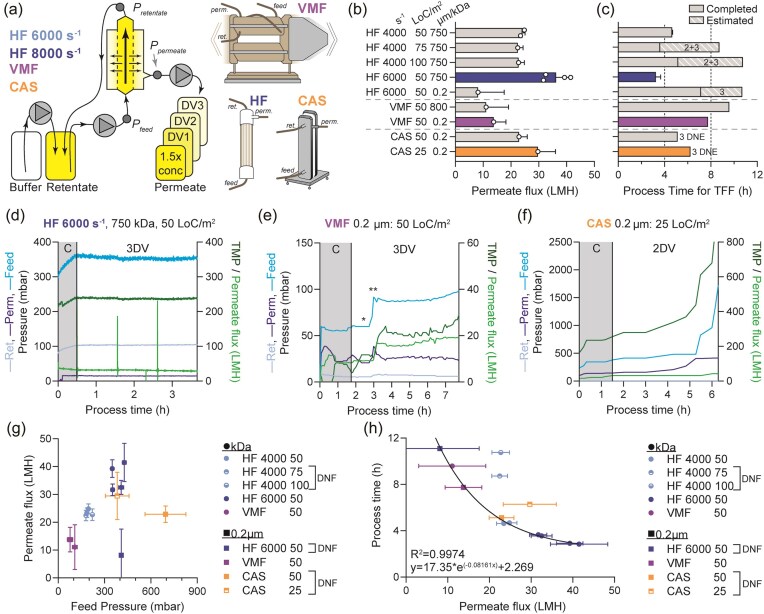
(a) Schematic of tangential flow filtration for cell wash: with permeate control pump, separate permeate fraction collection. A representative sketch of each of the three single-use membranes is depicted, where the feed, retentate (ret.), and permeate (perm.) lines are indicated. The VMF holder vibrates horizontally while in use. (b) Average permeate flow rate, flux expressed as L/m^2^/h (LMH), for each parameter tested. Conditions that permitted the highest flux per TFF modality is shown with coloured bars. Hatched bars indicate did not finish (DNF) runs terminated after 1 or 2 diavolume (DV) due to extended run time (as observed for HF) or increasing feed pressure (as observed for CAS), and the estimated run time was extrapolated. For HF, two shear were assessed: 4,000 and 6,000 s^−1^. Loading is expressed as litres of culture per membrane area squared (LoC/m^2^). Membrane pore sizes tested: 750 kDa, 800 kDa, and 0.2 µm. (c) Total processing time for each parameter tested. Solid bars indicate the measured length of processing time for 1.5x concentration followed by 3 DV. Hatched bars indicate the estimated time to complete the runs that were terminated after 1 or 2 DV due to low but stable permeate flux, and projected completion time is shown as an estimate based on completed DV fraction(s). In cases where the membrane fouled (CAS), the length of time to process the third DV was not estimated, and marked as ‘DNE’ (does not exist). Y-axis labels in (b) apply to (c) as well. (d) In-process pressure and flow rate measurements for best HF parameters. (e) In-process pressure and flow rate measurements for best VMF parameters. Feed flow increased from 561 (marked with asterisk, *) to 814 LMH (marked with two asterisks, **) to increase feed pressure and therefore permeate flux. (f) In-process pressure and flow rate measurements for the better of two conditions tested for CAS. Loading <25 LoC/m^2^ recommended. (g) Summary of TFF modality effect on feed pressure (mbar) and permeate flux (LMH). (h) Summary of effect of permeate flux on process time. Runs that did not finish (DNF, marked in legend) were excluded from regression analysis. *Abbreviations*: DV = diavolume; DNF = did not finish; TMP = transmembrane pressure (mbar).

**Fig. 2.**
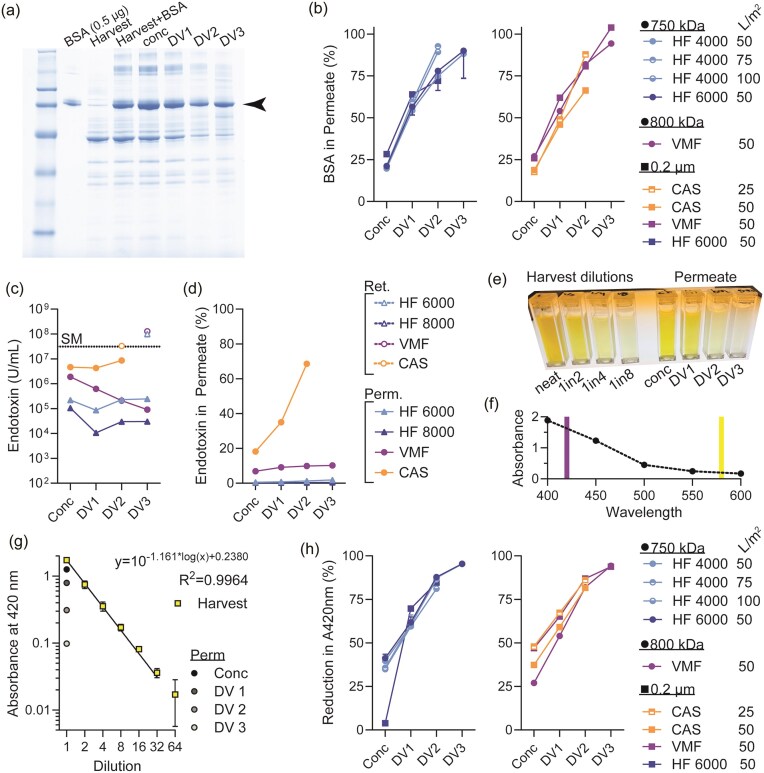
(a) Representative SDS-PAGE for BSA recovery from harvest and permeate fractions. (b) Quantitation of SDS-PAGE results for BSA transmission to permeate during TFF. Data split over two plots to improve clarity due to similarity of results. (c) Endotoxin concentration in starting material (SM), separate permeate fraction, and final retentate. Legend in (d) applies to (c). (d) Endotoxin in permeate as a percentage of total endotoxin in SM (representing 100%). (e) Representative cuvettes used for measuring yellow colour absorbance at 580 nm. (f) Absorbance of harvest supernatant solution scanned throughout visible wavelengths of light. Buffer used as blank for every wavelength. Violet and yellow lines indicate the regions for the wavelengths of these colours; 420 nm and 580 nm, respectively. (g) Linearity of absorbance at 420 nm (A_420nm_) using harvest supernatant dilution series, from two independent fermentation runs is shown. Representative permeate samples are shown and were measured undiluted. (h) Quantitation of A_420nm_ for each permeate fraction, expressed as percent reduction of harvest supernatant. Data split over two plots to improve clarity due to similar results. *Abbreviations*: BSA = bovine serum albumin; Perm = permeate.

**Fig. 3.**
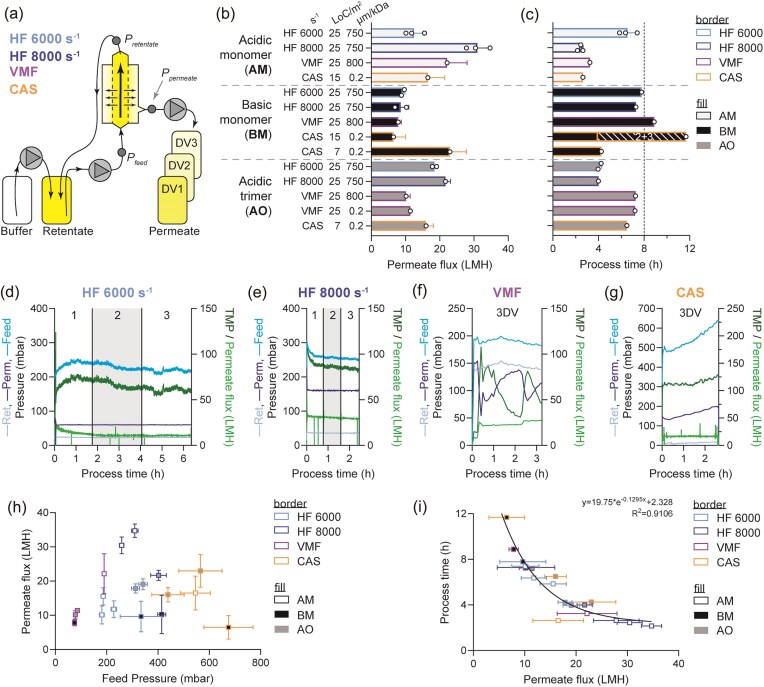
(a) Schematic of tangential flow filtration for clarification: with permeate control pump, separate permeate fraction collection. (b) Average permeate flow rate, flux expressed as L/m^2^/h (LMH), for each parameter tested. For HF, two shear rates were assessed: 6,000 and 8,000 s^−1^. Loading is expressed as litres of culture per membrane area squared (LoC/m^2^). Membrane pore sizes tested: 750 kDa, 800 kDa, and 0.2 µm. Legend in (c) applies to (b) as well. (c) Total processing time for each parameter tested. Solid bars indicate the measured length of processing time for 3 DV. Hatched bars indicate the estimated time to complete the run that was terminated after 1 DV due to low but stable permeate flux, and projected completion time is shown as an estimate based on completed DV fraction. Y-axis labels in (b) apply to (c) as well. (d–g) In-process pressure and flow rate measurements for 3DV collected using *E. coli* homogenate containing AM protein, with HF 6,000 s^−1^ (d), HF 8,000 s^−1^ (e), VMF (f), and CAS (g). HF and VMF with equivalent loading of 25 LoC/m^2^, and CAS with 15 LoC/m^2^. Membrane pore sizes tested for HF (750 kDa), VMF (800 kDa), and CAS (0.2 µm) listed here. X-axis length in all four panels are proportional to process duration. (h) Summary of TFF modality effect on feed pressure (mbar) and permeate flux (LMH). (i) Summary of permeate flux effect on process time, in the context of three different target proteins and three TFF modalities. No runs were excluded from regression analysis.

**Fig. 4.**
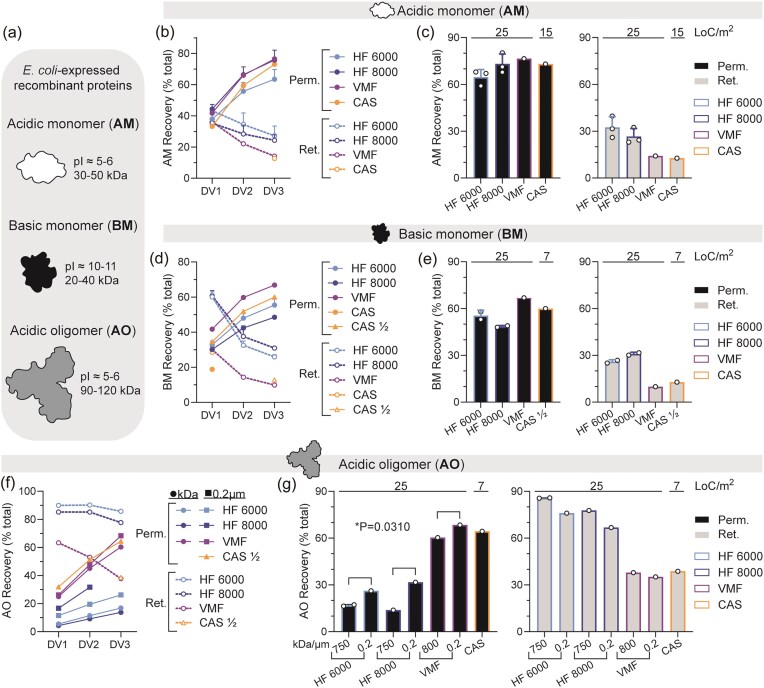
(a) Key physicochemical traits of three diverse recombinant proteins produced as *E. coli* expressed recombinant proteins. (b–g) These data are based on the experiments summarized in Fig. 3. Loading of *E. coli* homogenate is expressed as litres of culture per membrane area squared (LoC/m^2^). Loading for HF and VMF is 25 LoC/m^2^. Loading for CAS and CAS½ is 15 and 7 LoC/m^2^, respectively. (b) Upon completion of each diavolume (DV), the permeate and retentate were sampled for AM protein content. Cumulative recovery was assessed by RPLC and is expressed as a percent of starting total AM protein content. (c) Total AM recovery after 3DV, expressed as a percent of starting total AM protein content. (d) Upon completion of each diavolume (DV), the permeate and retentate were sampled for BM protein content. Cumulative recovery was assessed by RPLC and is expressed as a percent of starting total BM protein content. (e) Total BM recovery after 3DV, expressed as a percent of starting total BM protein content. (f) Upon completion of each diavolume (DV), the permeate and retentate were sampled for AO protein content. Cumulative recovery was assessed by RPLC and is expressed as a percent of starting total AT protein content. (g) Total AO recovery after 3DV, expressed as a percent of starting total AO protein content. *Abbreviations*: Perm. = permeate; Ret. = retentate.

This study used GraphPad Prism (version 10.1.2) for descriptive statistics. Comparison of fits was used for fitting curves in Figs. 1h and 3i, where one-phase exponential decay and quadratic regression analyses were assessed. Log-log line regression was used for Fig. 2d. A paired one-tail t-test was used for comparative analysis of pore size in Fig. 4g. Tukey's multiple comparisons test was used to analyze permeate samples shown in Fig. S1B and C. *p*-values, regression equations, and R^2^ statistics are displayed in respective figure panels.

## Results

### E. Coli Cell Concentration and Wash

Fermenter crude harvest material consisting of high-density *E. coli* was used for cell wash microfiltration studies, using automated systems for all three TFF modalities; HF, VMF, and CAS (Fig. 1a). Positive permeate pressure control was used to have better control of membrane polarization and minimize shear stress, as previously reported (Stressmann & Moresoli, 2008). The physical characteristics of the high-density *E. coli* material are described in Table 1. Higher parallel throughput was possible for the HF system based on equipment availability, therefore a wider range of conditions were first tested with HF. HF membrane loading of 50, 75, and 100 L of culture per m^2^ (LoC/m^2^) were evaluated, representing a CWW load of 5.7, 8.5, and 11.3 kg/m^2^, respectively. However, the 75 and 100 LoC/m^2^ HF runs were unable to be completed within 8 hr and were not pursued further (Fig. 1b and c). The ideal set of conditions for HF that promoted the highest permeate flux and lowest processing time were a shear of 6,000 s^−1^, membrane loading of 50 LoC/m^2^, and membrane pore size of 750 kDa (Fig. 1b and c). Four independent replicate runs were performed to verify the robustness of this result. This combination of parameters led to minimal decreases in permeate flux over the course of the experiment (Fig. 1d), leading to a highly consistent processing time of 4 hr (Fig. 1c). Interestingly, the same loading and shear conditions with a 0.2 µm pore-size membrane resulted in the worst HF outcome observed, suggesting that subtle optimization can have a large impact on HF process outcome (Fig. 1b and c). While 0.2 µm pore-size HF has been used to concentrate dilute *E. coli* cells, a shear rate of 27,000 s^−1^ was required to prevent ‘*bacterial attachment on membrane surfaces*’ (Zuponcic et al. 2024), which may exceed safe operating pressures for much higher density materials. Overall, the permeate flux rates are generally within typical TFF microfiltration operating ranges previously reported (Stressmann & Moresoli, 2008). As the maximum membrane loading for HF was 50 LoC/m^2^ (8.5 CWW kg/m^2^), VMF and CAS were compared to this standard. VMF could process the equivalent membrane load, with the 0.2 µm PES membrane providing a slight advantage in shorter processing time and higher permeate flux, compared to the 800 kDa PVDF membrane (Fig. 1b and c). In fact, the feed pressure was initially so much lower than all other conditions, that raising it could enable a doubling of permeate flux (Fig. 1e). In contrast, CAS was unable to complete the prescribed 3 diavolume (DV), being terminated after 2 DV with both 50 and 25 LoC/m^2^ load, resulting in membrane fouling (Fig. 1f). Loading <25 LoC/m^2^ (<2.8 CWW kg/m^2^) decreases industrialization capacity, and so further optimization with the CAS system was not pursued for cell wash microfiltration. The results of these TFF modalities are not directly comparable, due to product characteristics that will be more fully compared in the section Discussion. To summarize the cell concentration and wash studies operating parameters, higher feed pressures were positively correlated with higher permeate flux, and higher permeate flux was positively correlated with a shorter processing time (Fig. 1g). Overall, HF with optimal conditions resulted in the shortest processing time. However, there remains interest in increasing VMF permeate flux to assess its impact on processing time.

While shorter process time is a key benefit in the bioprocessing industry, the efficiency of the cell wash also requires assessment. As fermentation progresses, the spent media (i.e. supernatant) accumulates metabolic by-products and waste (Korz et al. 1995), and changes from clear to yellow in colour. The removal of these waste products through buffer exchange often improves subsequent purification steps. Therefore, BSA was spiked-in to the starting material and the permeate was assessed for the clearance of BSA during concentration and DF (Fig. 2a). Strikingly, BSA clearance was 100% for VMF after 3DV, as opposed to HF conditions which could achieve an average of 90% BSA clearance (Fig. 2b). BSA clearance via VMF was higher for the 0.2 µm membrane than the 800 kDa membrane, which is advantageous as this pore size also resulted in a shorter processing time. Similarly, the removal of endotoxin and yellow pigment from the spent media was assessed using the starting material supernatant and permeate samples. Potential endotoxin removal from the crude harvest material was similarly assessed for all modalities (Fig. 2c). Represented as a percent of total endotoxin, the cell wash microfiltration parameters for HF and VMF could not significantly reduce the amount of endotoxin (Fig. 2c). Some endotoxin removal from the crude harvest material was observed for CAS. However, the membrane fouled prematurely at the second diavolume (DV), and the CAS runs could not be completed. To assess the yellow pigment reduction from spent media (Fig. 2e), the pigment absorbed most strongly in the violet regions of visible light (Fig. 2f), as expected. Given a strong linear correlation observed for a dilution series of crude harvest supernatant (Fig. 2g), the reduction of yellow pigment could be quantitatively assessed as a percent of the starting material. Here, all modalities performed similarly at each stage of microfiltration, with all completed runs achieving a >95% reduction in yellow pigment (Fig. 2h). These data collectively suggest that 1.5-fold concentration and 3-diavolume filtration are ideal conditions for high-density *E. coli* cell wash microfiltration, regardless of the TFF modality chosen.

### E. Coli Lysate Clarification

High-density *E. coli* was lysed using high pressure homogenization and used for lysate clarification studies, using automated systems with positive permeate pressure control for all three TFF modalities; HF, VMF, and CAS (Fig. 3a). Cell lysis generated viscous solutions containing cell debris, DNA, *E. coli* HCPs, and other impurities, in addition to the recombinant target protein. The physical characteristics of the lysate material are described in Table 2. This material was twice the density of the 40–60 g/L that was previously reported (Bailey et al., 1997), based on initial CWW, for lysate material that was otherwise similarly homogenized. Clarification studies of *E. coli* lysate were assessed for process performance, and then for target protein recovery of an AM, BM, or AO. Two initial conditions were tested prior to the study launch. Preliminary buffer screening experiments for the BM protein indicated that the conductivity needed to be raised to 500 mM NaCl to improve membrane transmission during lysate clarification (Fig. S1A). With both HF and VMF modalities, this increased processing time (Fig. S1A) is likely attributable to the increase in solution viscosity (Table 2). Secondly, a technical limitation was the ability to run only one VMF or CAS experiment at a time, whereas in contrast, the operating system for HF could run up to eight parallel runs simultaneously. Since only one VMF or CAS experiment could be run at a time, the starting material needed to be preserved in such a way that all runs could be fairly compared. Therefore, the permissibility of thawed homogenate was assessed, as it would allow parallel studies with frozen aliquots of the same starting material. Fresh and thawed homogenate containing AM protein were tested at two different shear rates by HF. No significant differences in either total protein transmission through the membrane (Fig. S1B), or target protein recovery (Fig. S1C) were observed. Therefore, all subsequent experiments were performed from frozen aliquots of lysate starting material, each containing one of the AM, BM, or AO target proteins, which were thawed on the day of use.

Similar to the cell wash microfiltration studies, the effect of TFF modality, membrane loading, and HF shear rate were evaluated for the permeate flux and process duration of 3DV for lysate containing one each of the three target proteins (Fig. 3b and c). HF and VMF were equivalently loaded at 25 LoC/m^2^ (2.8 kg lysed *E. coli* CWW per m^2^) and processing time was generally between 4 and 8 hr. Based on the lower loading required for CAS during cell wash microfiltration studies, lower loadings of 15 and 7 LoC/m^2^ (0.8 and 1.7 kg lysed *E. coli* CWW per m^2^) were assessed with the goal of determining the maximal load possible while also maintaining a processing time <8 hr. Overall, the permeate flux rates were generally within typical TFF microfiltration operating ranges previously reported (Stressmann & Moresoli, 2008). The higher fluxes (shorter process times) were observed for lysate-containing AM, whereas the opposite was observed for the higher salt-containing BM lysate, which had lower permeate flux (longer process times; Fig. 3b and c). This is potentially attributable to the higher viscosity and salting-out properties of the BM lysate solution, coupled to potential non-Newtonian effects on shear thinning (Table 2, Tsumoto et al., 2007; Zhang et al., 2022). Similar to previous reports (Bailey et al., 1997), permeate flux modestly decreased over the course of the HF runs (Fig. 3d and e). In contrast, permeate flux for VMF could be increased during lysate clarification (Fig. 3f). In brief, feed and retentate pressure tended to be consistent during operation, while it was observed that permeate pressure gradually increased, leading to an overall decrease in TMP. By increasing permeate flux, this allowed for faster operation that did not significantly decrease permeate pressure. The CAS runs were uniformly operated at a lower membrane loading, as pressures increased in all trials (Fig. 3g), and a solid load of <15 LoC/m^2^ (<1.7 kg lysed *E. coli* CWW per m^2^) was needed for uniform CAS-based lysate clarification. To summarize the lysate clarification studies operating parameters, feed pressures are associated with the TFF modality; high, medium, and low pressures were associated with CAS, HF, and VMF, respectively (Fig. 1h). However, there was no clear relationship of feed pressure to permeate flux. Like the cell wash microfiltration studies (Fig. 1h), a strong nonlinear relationship was observed between permeate flux and process duration (Fig. 3i). The results of these TFF modalities are not directly comparable, due to product characteristics that will be more fully compared in the section Discussion. However some trends could be observed, such as membrane loading for HF and VMF were equivalent and both out-performed CAS for lysate clarification. Processing time for VMF was generally between the times for HF at two different shear rates. A higher shear rate led to shorter processing time in low salt conditions (AM, AO), whereas it appeared to be detrimental for high-salt solutions (BM).

While process time and membrane load are important factors for process control and industrialization, target protein recovery is the key measure. Diversity of target proteins was critical for assessment of the potential for a near-universal TFF modality, therefore proteins of different size and charge were tested (Fig. 4a). Each protein sample was quantified by reverse phase liquid chromatography to evaluate target protein recovery at each stage (Fig. S2). Using the same experiments shown in Fig. 3, target protein recovery of AM was assessed by RPLC from each DV fraction and expressed cumulatively (Fig. 4b). AM recovery from lysate was highest for HF (8,000 s^−1^: 73 ± 6%) and VMF (77%) at equivalent loading of 25 LoC/m^2^ (Fig. 4c). Overall, the mass balance of AM target protein (i.e. sum of permeate and retentate fractions) was 93 ± 6% of starting material (i.e. close to 100%), suggesting low AM loss to the membrane across all modalities.

Next, BM target protein recovery was assessed by RPLC from each DV fraction and expressed cumulatively (Fig. 4d). BM target protein recovery after 3DV, from high-salt-containing lysate solution, was markedly highest for VMF (67%), compared to the next highest condition of HF (6,000 s^−1^: 55 ± 4%), at equivalent membrane loading (Fig. 4e). While a BM recovery percentage of 60% using CAS was observed at 7 LoC/m^2^, this loading represents less than one third of the total recovery achieved by VMF at 25 LoC/m^2^, and therefore does not offer high industrialization potential. Overall, the mass balance of BM target protein (i.e. sum of permeate and retentate fractions) was 78 ± 4% of starting material, suggesting some form of BM loss to the membrane was likely across all modalities.

Finally, AO target protein recovery was assessed by RPLC from each DV fraction and expressed cumulatively (Fig. 4f). AM target protein recovery after 3DV was low when using membranes with 750 and 800 kDa pore sizes (Fig. 4g). Since AO is larger than both AM and BM, larger membrane pore sizes were assessed for improving AO transmission. This has been similarly reported for *E. coli* lysate HCPs exceeding 80 kDa in size when using a HF membrane with 0.1 µm pores (Bailey et al., 1997). Indeed, membranes with 0.2 µm pores were significantly better for AO recovery for both HF and VMF (*p = .0310). However, recovery with VMF (68%) far outpaced the next highest condition's recovery of HF (8,000 s^−1^: 32%), at equivalent membrane loading (Fig. 4e). While AO recovery using CAS was 64% with 7 LoC/m^2^, again this loading represents less than one third of the total recovery achieved by VMF at 25 LoC/m^2^. Overall, the mass balance of AO target protein (i.e. sum of permeate and retentate fractions) was 100 ± 3% of the starting material, suggesting low AO loss to the membrane across all modalities.

**Fig. 5.**
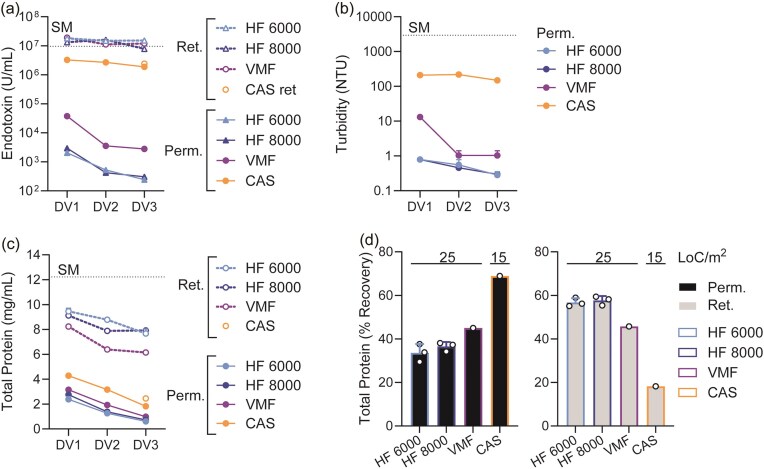
(a–c) Upon completion of each diavolume (DV), the permeate and retentate were sampled for endotoxin (a), turbidity (b), and total protein (c) content. These data are based on the experiments summarized in Fig. 4b and c, where all four main TFF operating conditions were the most similar in terms of process time, homogenate load per membrane surface area, and target protein recovery. (d) Total protein recovery, consisting of *E. coli* host cell proteins mixed with target protein, is shown after 3DV.

Cumulative recovery of target proteins at 3DV achieves a good balance for maximizing target protein recovery while maintaining the process time within a reasonable range (Fig. 4b and d). To further characterize the utility of 3DV as the unit operation setting for lysate clarification, reductions in unwanted material were assessed: endotoxin, particles, and protein (including recombinant target protein and HCP, inferred by total protein quantification). A >4 log-fold reduction of endotoxin concentration was observed in the third DV fraction of VMF and HF, compared to the *E. coli* lysate starting material (Fig. 5a). Similarly, particle concentration was reduced from 3 450 ± 540 to ≤1 NTU by the third DV fraction of VMF and HF (Fig. 5b). Lastly, total protein membrane transmission into the permeate was assessed for each permeate fraction (Fig. 5c). Cumulative recovery of these 3DV showed that VMF and HF could remove the majority of total protein (Fig. 5d).

Overall, HF (with a shear-dependent effect) and VMF appear to be the best modalities for both microfiltration of high-density *E. coli* cells and high-density lysate. HF is generally faster with a slightly lower target protein yield, while VMF is slightly slower but achieves a higher target protein yield. Cell debris is considered highly likely to cause membrane fouling due to physical properties (Table 2, Besnard et al., 2016). Therefore, VMF with its lower flow rates and pressures (Fig. 1g and 3h), may be advantageous for process robustness. These trends apply to all three diverse recombinant proteins expressed using high-density *E. coli* fermentation. Throughout the assessment of TFF modalities, transmission of undesirable endotoxin, cell particles, and total protein were systematically higher with CAS. It is unclear whether this is due to the lower membrane loading required to reduce fouling, or to some of the membrane's characteristics. As CAS TFF requires further optimization for high-density *E. coli* lysate clarification, it was not considered further for the potential to be a near-universal TFF modality.

## Discussion

A key consideration for future development of microfiltration processes is the scalability of the two TFF modalities; HF and VMF. Nonlinear scalability of HF for clarification has been reported (Besnard et al., 2016), but not currently evaluated. Solutions with higher suspended solid content or viscosity (i.e. high-density *E. coli* cell culture and lysate) may contribute to a greater pressure drop along the membrane. HF membrane effective length may need to be constrained to prevent this pressure drop, otherwise TMP (i.e. shear rate) is increased to compensate. Pressure drop and potential non-Newtonian effects on shear thinning (Zhang et al., 2022) may contribute to HF's nonlinear scaling, especially since membrane length is necessarily increased at the industrial scale. Purported linear scaling of VMF remains a topic of interest. Recent studies have reported the capacity of VMF to concentrate microalgal cultures, at the 25 L and 170 L scale, down to the consistency of a slurry (Grácio et al., 2024; Six et al., 2024). Furthermore, variability in cell culture outcomes could impact TFF performance, especially in cases where batch-to-batch variability is high. Similar studies are warranted to evaluate the present study's cell wash microfiltration and lysate clarification operating parameters at a larger scale.

The present study used multiple membrane chemistries and pore sizes, so results are not directly comparable across each modality due to vendor supply constraints. However, polyvinylidene fluoride (PVDF), modified polyether sulfone (mPES), and regenerated cellulose (RC) membranes are all established materials for microfiltration applications in the biopharmaceutical industry (Stressmann & Moresoli, 2008). All membranes are generally considered to be low fouling, low in protein absorption, and hydrophilic. Indeed, equivalent low protein absorption was observed for all TFF modalities for the AM and AO lysate clarification studies (i.e. permeate and retentate fractions sum to 100% of the starting material). In contrast, BM protein content in the permeate and retentate fractions was observed to be 78 ± 4% of the starting material, suggesting potential membrane binding. However, as this was observed for all TFF modalities and membrane chemistries, it is likely due to the effects of high salt that pertained to the BM studies (Tsumoto et al., 2007). Other HF lysate clarification studies have compared PVDF (used in the present study) to PES and cellulose acetate (not used in present study) and have found performance differences (Bailey et al., 1997). A notable key limitation of membrane chemistry comparability is the difference in extractable and leachables (Kushwah et al., 2022). PES, PVDF, and cellulose have known and distinct extractables and leachables profiles. Additionally, total organic carbon can be reduced in part by rinsing membranes (Menzel et al., 2022). However, some membrane chemistries may have varying suitability for the pharmaceutical industry based on these profiles and application-specific uses.

Cost of goods is another consideration when comparing multiple single-use TFF technologies. Single-use technology can reduce production costs associated with cleaning and sterilization validation, shorten batch turn-around times, reduce plant footprint and capital investments, as well as reduce the risk of product cross-contamination (Ottinger et al. 2022). However, each production run requires a new single-use unit. To compare across the three modalities tested, the cost of each modality per 100 cm^2^ of membrane surface area is reported from highest to lowest: 514 USD for HF, 304 USD for VMF, and 150 USD for CAS. While CAS is the most economical option per membrane surface area, this modality was unable to match the high solid loading of the other two TFF modalities. Therefore, at equivalent loading, CAS would require approximately three times more surface area to produce similar results as the HF and VMF in this study, therefore the adjusted cost is 450 USD. While all three TFF modalities require an automated system for feed pump control, VMF requires one additional source of power for vibration. A standard electrical outlet (120 V, 15 A) is sufficient to provide the vibrational power for the Lab280 to perform TFF. The vibration frequency is 20–23 Hz. Overall, VMF is the most economical option in this study. However, all three single-use TFF modalities are relatively similar in cost.

## Conclusion

Overall, this study identified optimal operating conditions for both cell wash microfiltration and lysate clarification of high-density *E. coli* culture material. In both applications, HF and VMF achieved primary outcomes and have the capacity to be a near-universal TFF modality for midstream unit operations, especially at lab scale (<10 L). Both HF and VMF systems are fully automated and permit overnight operation with minimum supervision. This approach has broad applications in the manufacture of microbial enzymes and other proteins across the food, detergent, pharmaceutical, and paper industries (Raveendran et al., 2018). This study may serve as a guide upon further intensification of eukaryotic mid- to low-density production of biologics, and in emerging fields such as recombinant protein production in *Vibrio natriegens* (Smith et al., 2024), which may require development to move beyond centrifugation for industrialization.

## Supplementary Material

kuaf008_Supplemental_Files

## Data Availability

Data available on request.
