# Supplementary figures and images for: Exploration of alternative microfiltration modalities for the harvest and clarification of diverse recombinant proteins from high-density E. coli culture and lysate using hollow fibre, flat sheet cassette, and vibro membrane filtration technologies
Source: J Ind Microbiol Biotechnol. 2025 Apr 4;52:kuaf008. doi: 10.1093/jimb/kuaf008 (PMC12022607; doi:10.1093/jimb/kuaf008)

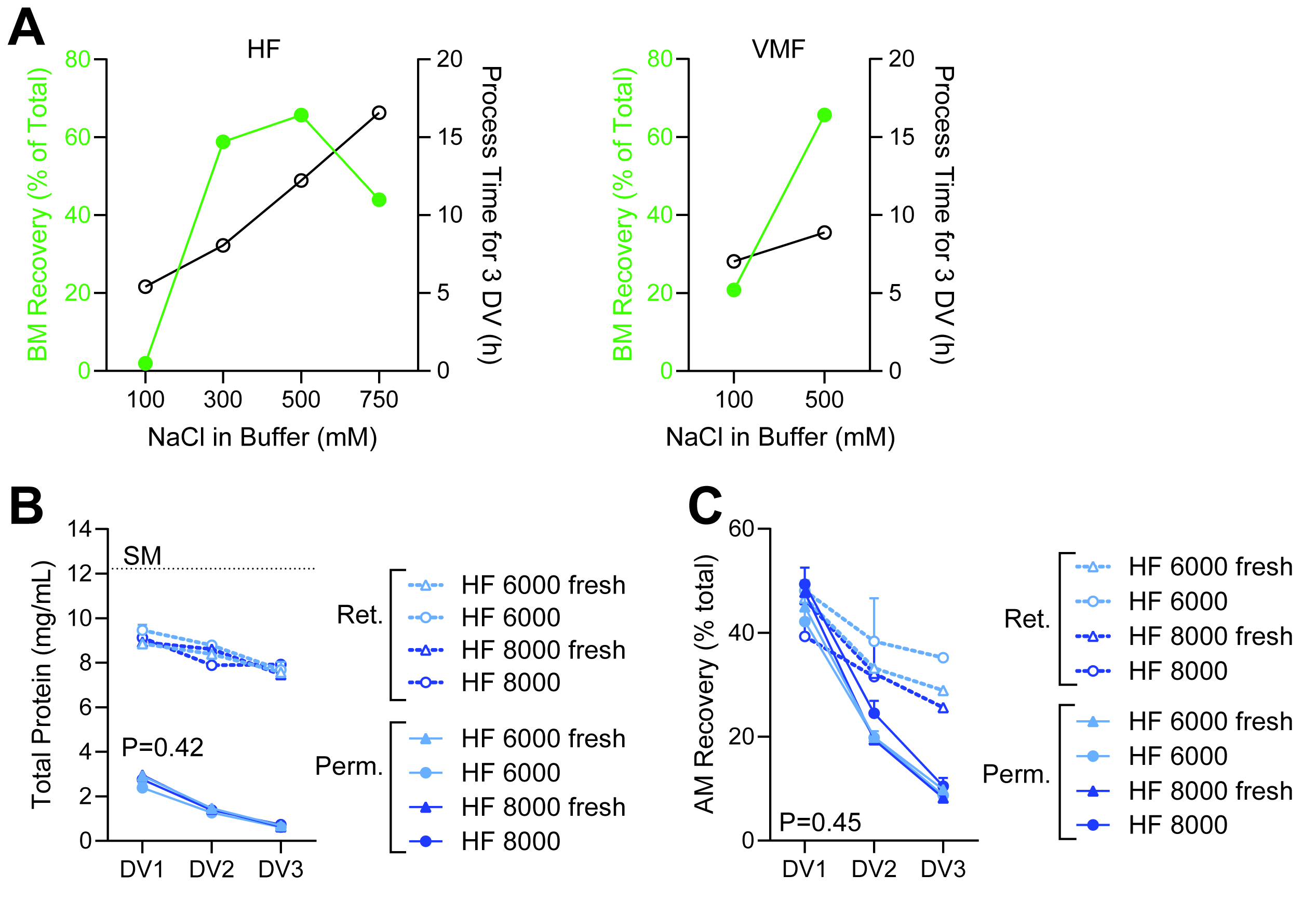

Supplement: kuaf008_Supplemental_Files [file kuaf008_supplemental_files.zip › Figure S1.tif]
